# HACANCOi: a new H^α^-detected experiment for backbone resonance assignment of intrinsically disordered proteins

**DOI:** 10.1007/s10858-020-00347-5

**Published:** 2020-10-28

**Authors:** Mikael Karjalainen, Helena Tossavainen, Maarit Hellman, Perttu Permi

**Affiliations:** 1 Department of Chemistry, Nanoscience Center, University of Jyväskylä, Jyväskylä, Finland; 2 Department of Biological and Environmental Science, University of Jyväskylä, Jyväskylä, Finland

**Keywords:** *E.coli*, EspF, GB1, Intrinsically disordered protein, IDP, Resonance assignment, SNX9 SH3

## Abstract

Unidirectional coherence transfer is highly efficient in intrinsically disordered proteins (IDPs). Their elevated ps-ns timescale dynamics ensures long transverse (T_2_) relaxation times allowing sophisticated coherence transfer pathway selection in comparison to folded proteins. ^1^H^α^-detection ensures non-susceptibility to chemical exchange with the solvent and enables chemical shift assignment of consecutive proline residues, typically abundant in IDPs. However, many IDPs undergo a disorder-to-order transition upon interaction with their target protein, which leads to the loss of the favorable relaxation properties. Long coherence transfer routes now result in prohibitively large decrease in sensitivity. We introduce a novel 4D ^1^H^α^-detected experiment HACANCOi, together with its 3D implementation, which warrant high sensitivity for the assignment of proline-rich regions in IDPs in complex with a globular protein. The experiment correlates ^1^H^α^_i_, ^13^C^α^_i_, ^15^N_i_ and $^{13} C^{\prime}_{i}$ spins by transferring the magnetization concomitantly from ^13^C^α^_i_ to ^15^N_i_ and $^{13} C^{\prime}_{i}$. The B1 domain of protein G (GB1), and the enteropathogenic *E.* *coli* EspF in complex with human SNX9 SH3, serve as model systems to demonstrate the attainable sensitivity and successful sequential assignment.

**Electronic supplementary material:**

The online version of this article (10.1007/s10858-020-00347-5) contains supplementary material, which is available to authorized users.

## Introduction

Intrinsically disordered proteins (IDPs), proteins with intrinsically disordered regions (IDRs) or modular proteins containing intrinsically disordered linkers (IDLs) have raised great interest in the scientific community in the last two decades. Not only because these proteins or regions have been tightly linked to disease related biology, but also because they have significantly influenced our perception of mechanistic structural biology in general. The fundamental difference between the classical folded protein kingdom and their disordered rivals, often referred to as the ‘dark proteome’ calls for different means to study them using NMR spectroscopic tools.

Whereas prohibitively fast transverse relaxation is the biggest hurdle in the structure determination of larger folded proteins and complexes, resonance overlap constitutes the major hindrance in the chemical shift assignment and hence structural characterization of disordered systems. Resonance overlap substantially limits the feasibility of the well-established amide proton (H^N^) detection based experiments which rely on C^α^/C^β^ shifts to obtain sequential connections, commonly used for the assignment of folded proteins (for reviews see e.g. Sattler et al. 1999; Permi and Annila 2004). Even though amide signals in a ^1^H, ^15^N HSQC of an IDP can show fair dispersion, residue type dependent ^13^C^α^ and ^13^C^β^ clustering strongly reduces the applicability of these experiments. Carbonyl carbons, however, show greater dispersion thanks to their larger sensitivity to the type of neighboring residues in the primary sequence, and therefore better meet the demands of assignment (Yao et al. 1997; Mäntylahti et al. 2009; Bermel et al. 2012). Combination of ^13^C′ dispersion with the high sensitivity and robustness of the H^N^-detection based experiments, additionally exploiting their compatibility with the TROSY and BEST implementations (Solyom et al. 2013; Brutscher et al. 2015), has been a successful work-around scheme for the assignment of many disordered systems. These include experiments which exploit increase of dimensionality from conventional 3D to 4-7D spectra (Fiorito et al. 2006; Motackova et al. 2010; Nováček et al. 2011; Kazimierczuk et al. 2013; Brutscher et al. 2015; Yoshimura et al. 2015). Recently we and others have proposed H^N^-detected experiments which allow bridging the gaps in amino acid sequence fragments due to single prolines in PXP or XPX moieties (Liu and Yang 2003; Hellman et al. 2014; Tossavainen et al. 2020).

The H^N^/C′ approach is, however, less optimal for studies at neutral or alkali sample pH and/or proteins that contain a high percentage of prolines. To this end, two different approaches have been proposed; the direct ^13^C, especially ^13^C′, detection based experiments (Bermel et al. 2006a, 2009, 2012) and the ^1^H^α^ based detection (Mäntylahti et al. 2010, 2011). The direct detection of ^13^C′ spins instead of detection of H^N^ renders the assignment non-susceptible to chemical exchange with the solvent at pH higher than 7. In addition, this approach enables the assignment of consecutive proline residues and is therefore suitable for studying proline rich IDRs/IDLs. However, to fully reclaim the advantages of ^13^C-detection, a probehead having ^13^C as the inner coil is required as an inherent sensitivity loss by a factor of eight, owing to the S/N = γ_H_^3/2^/γ_C_^3/2^ dependence on sensitivity, applies in comparison to ^1^H-detection. Showalter and co-workers have evaluated that sample concentration with the ^13^C-detection approach should not fall significantly below 500 μM when using three-dimensional ^13^C-detected experiments for chemical shift assignment (Bastidas et al. 2015).

Similar to its ^13^C-detected counterpart, the ^1^H^α^-detection based assignment strategy is per se insensitive to chemical exchange with the solvent as ^1^H^α^s are considered as non-labile protons irrespective of pH. Also, assignment of consecutive prolines can readily be accomplished, making these experiments attractive alternatives to ^13^C-detection based pulse schemes (Mäntylahti et al. 2010, 2011; Permi and Hellman 2012; Tossavainen et al. 2020). Moreover, unless a ^13^C-detection optimized probehead is used, these experiments offer superior sensitivity with respect to ^13^C-detected experiments (Wong et al. 2020), therefore enabling significant time savings. Previously we have successfully used a set of three 3D ^1^H^α^-detected experiments, i.e. iHA(CA)NCO, HA(CA)CON and (HACA)CON(CA)HA in the assignment of several IDPs and proline-rich IDLs (Hellman et al. 2014; Aitio et al. 2012; Tossavainen et al. 2020). Extension to 4D, by utilizing ^13^C^α^ chemical shifts, facilitated the assignment of 161-residue BilRI, bacterial interleukin receptor I, from an opportunistic oral pathogen *A.* *actinomycetemcomitans*, half of whose amino acid sequence is composed of three residues only, that is alanines (23%), lysines (14%) and aspartic acids (13%) (Tossavainen et al. 2020).

Indeed, the unidirectional coherence transfer is highly efficient in IDPs. Thanks to their elevated ps-ns timescale dynamics IDPs have long transverse relaxation times (T_2_s) allowing more sophisticated coherence transfer pathway selection in comparison to globular proteins. In ^1^H^α^-detected experiments this can further be boosted by measuring spectra at an elevated temperature, 35–45 °C, as temperature dependent exchange rate of labile amide protons with solvent is not a problem with ^1^H^α^-detected experiments. The coherence transfer efficiency of the unidirectional experiments, however, drops dramatically in the case of folded proteins, or when an IDP or IDR/L folds upon binding when interacting with its target. In the latter case, favorable relaxation properties are partially lost at least for the residues directly and sometimes indirectly involved in binding. Under these conditions, the long coherence transfer routes become a liability resulting in prohibitively large losses in sensitivity in these experiments. In this manuscript, we introduce a novel 4D (3D) ^1^H^α^-detected experiment HACANCOi, which warrants high sensitivity for the assignment of proline-rich regions in IDPs in complex with a globular protein.

## Materials and methods

### Cloning, expression, and purification of SNX9 SH3 and EspF

The gene encoding the SH3 domain (residues 1–64) of human SNX9 (Sorting nexin 9) (UniProt Q9Y5X1) (SNX9 SH3) was cloned to pET15b vector (Novagen) into the NdeI and XhoI sites. The gene encoding residues 115–161 of EspF (UniProt B7UM88) (EspF) was cloned to pET15b vector with N-terminal GB1 fusion protein into the NdeI and XhoI sites. TEV protease (from Tobacco Etch Virus) cleavage site was added between GB1 fusion protein and EspF. Both protein constructs carried the N-terminal His-tag. All the genes were synthetic, obtained from GenScript Inc., USA.

Production of ^15^N and ^13^C labeled or unlabeled SNX9 SH3 or EspF proteins was carried out by transforming plasmids into the BL21(DE3) cells. The cells were grown in M9 minimal media, supplemented with 1 g/l of ^15^NH_4_Cl and 2 g/l ^13^C-D-glucose as the sole nitrogen or nitrogen and carbon source, respectively, or in LB media for obtaining unlabeled proteins. Cell culture was incubated at 37 °C and the temperature was decreased to 16 °C when OD of the cell culture reached 0.4 and protein production was induced with 1 mM IPTG when OD of the cell culture reached 0.6. The cells were further incubated at 16 °C for 16 h and collected by centrifugation. The cells were disrupted with sonication and the resulting supernatant was clarified by centrifugation with 30,000×*g*.

The clarified supernatant of His-Tagged SNX9 SH3 and His-Tagged GB1-EspF fusion protein was applied to the 1-mL His GraviTrap column (GE Healthcare) according to the manufacturer’s instructions. Imidazole was removed from eluted proteins by PD-10 (Ge Healthcare) before protease cleavage.

The His-Tag from the SNX9-SH3 was removed by thrombin protease and from the His-Tag-GB1-EspF fusion by TEV protease digestion. Digestion mixtures were applied to His GraviTrap column. Cleaved EspF and SNX9 SH3 eluted with flow-through, that were concentrated and applied into the Superdex75 (16/60) or Superdex30 (16/60) gel filtration column, respectively. Columns were equilibrated with NMR buffer (20 mM sodium phosphate pH 6.5, 50 mM NaCl). Fractions, containing purified proteins, were pooled and concentrated by Vivaspin2 (SartoriusStedim). All the gel filtrations were performed by using the ÄKTA Purifier FLPC purification system (GE Healthcare).

### NMR spectroscopy

Two-dimensional iHA(CA)N(CO) and HA(CA)N(COi) experiments were measured at four different temperatures 5, 15, 25 and 35 °C using 2 mM ^15^N, ^13^C labeled B1 domain of protein G (GB1), dissolved in 95%/5% H_2_O/D_2_O sodium phosphate buffer, pH 5.5. Three/four-dimensional HA(CA)NCOi/HACANCOi as well as three-dimensonal HA(CA)CON experiment were acquired for a binary complex between 1.9 mM ^15^N, ^13^C labeled SNX9 SH3 and 1.5 mM 47-residue repeat of EspF and 2.5 mM unlabeled SNX9 SH3 and 1.5 mM ^15^N, ^13^C labeled EspF complex, respectively. All spectra were recorded on a Bruker AVANCE III HD 800 MHz spectrometer equipped with a TCI ^1^H/^13^C/^15^N cryoprobe. Traditional sampling was used for collecting two- and three-dimensional data whereas non-uniform sampling (NUS) with the NUS-sampler algorithm and random order sampling at density of 7% was employed for collecting four-dimensional data (Table 1). No sample point optimization, based on either T_2_ or *J* coupling values, was employed during the data collection. The NMR data were processed with Topspin 3.5 software package (Bruker Inc.) either using fast-Fourier transform or multi-dimensional decomposition (MDD) data processing for NUS data with non-recursive MDD and specifying constant-time dimension for t_3_. The reconstruction took roughly 2 h on a standard PC attached to the spectrometer. Motivation for using traditional sampling instead of NUS in 3D data was to warrant optimal S/N for the weakest, binding exchange broadened, cross peaks in the complex without sacrifying the resolution. Data analysis was accomplished using CcpNmr analysis v. 2.4.2 (Vranken et al. 2005). Chemical shifts and structural data of the SNX9-SH3 – EspF complex will be published elsewhere.

**Table 1:** Data acquisition parameters

	Number of complex points (acquisition time)						
Experiment	F_1_ (ms)	F_2_ (ms)	F_3_ (ms)	F_4_ (ms)	nD	Number of scans	Experimental time
iHA(CA)N(CO)	200 (36.2) ^15^N	-	-	256 (53.3) ^1^H^α^	2	32	1 h 20 m
HA(CA)N(CO)i	200 (36.2) ^12^N	-	-	-	2	32	1 h 20 m
HA(CA)NCOi	64 (13.2) ^13^C’	80 (11.7) ^15^N	-	256 (53.3) ^1^H^α^	3	16	22 h
HA(CA)CON	80 (11.7) ^15^N	64 (13.2) ^13^C’	-	256 (53.3) ^1^H^α^	3	8	11 h
HACANCOi	80 (11.7) ^15^N	64 (10.2) ^15^N	48 (4.0) ^13^C^α^	256 (53.3) ^1^H^α^	4	8	23 h

## Results and discussion

Our group is actively working with IDPs harboring SH3 domain targeting short linear motifs (SLiMs). Classically SH3 domain interactions are mediated by proline-rich SLiMs. Due to the lack of an amide proton in the N-substituted proline residue, structural characterization of these epitopes using conventional H^N^-detected experiments is challenging. To overcome this limitation, we have employed a suite of 3D (or 4D) ^1^H^α^-detected experiments for the assignment of these proline-rich SLiMs (Mäntylahti et al. 2010, 2011; Permi and Hellman 2012; Tossavainen et al. 2020). Although this approach has been very successful for assigning several IDPs and their complexes (Aitio et al. 2010, 2012; Hellman et al. 2014; Tossavainen et al. 2017; Thapa et al. 2020), the sensitivity of these experiments may become an issue upon complex formation, stemming from a longer magnetization transfer delay needed for the unidirectional coherence transfer pathway selection. To this end, we have designed a novel 4D (together with its 3D implementation) ^1^H^α^-detected experiment, which offers significantly higher sensitivity in more slowly tumbling systems, e.g. IDPs in complex with structural domains, at the expense of data redundancy, that is exhibition of *intraresidual* and *sequential* *cross* *peaks* simultaneously in one experiment.

### The 4D HACANCO experiment

**Fig. 1:**
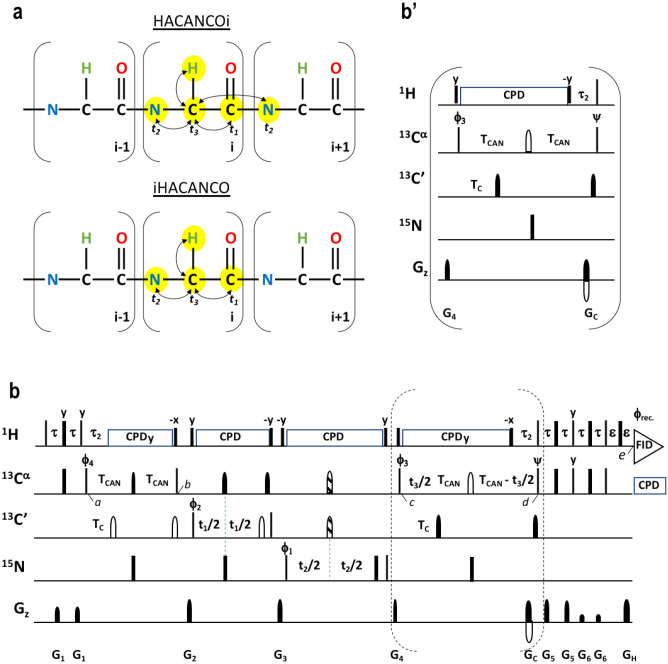
Description of the HACANCOi experiment. **a** Schematic presentation of magnetization transfer pathway during the proposed 4D HACANCOi experiment and 4D iHACANCO scheme (Mäntylahti et al. 2010; Tossavainen et al. 2020). Black arrows indicate the so-called out-and-back transfer pathway from ^1^H^α^(i) to ^13^C^α^(i) and further to ^13^C′(i) and ^15^N(i)/^15^N(i + 1). **b** 4D HACANCOi experiment to correlate ^1^H^α^(i), ^13^C^α^(i), ^13^C′(i) and ^15^N(i)/^15^N(i + 1) chemical shifts. Inset **b′** 3D HA(CA)NCOi experiment to correlate ^1^H^α^(i), ^13^C′(i) and ^15^N(i)/^15^N(i + 1) chemical shifts. Narrow and wide filled bars on ^1^H and ^15^N channels correspond to rectangular 90° and 180° pulses, respectively, applied with phase x unless otherwise stated. All ^13^C pulses are band-selective shaped pulses, denoted by filled narrow bars (90°) and filled and unfilled half ellipsoids (180°). Pulses denoted with unfilled bars are applied on-resonance. The ^1^H, ^15^N, ^13^C′, and ^13^C^α^ carrier positions are 4.7 (water), 121 (center of ^15^N spectral region), 174 ppm (center of ^13^C′ spectral region), and 54 ppm (center of ^13^C^α^ spectral region). The ^13^C carrier is initially set to the middle of ^13^C′ region (174 ppm), and shifted to ^13^C^α^ region (54 ppm) prior to 90° ^15^N pulse ϕ_3_. All band-selective 90° and 180° pulses for ^13^C^α^ (54 ppm) and ^13^C′ (174 ppm) have the shape of Q5 and Q3 (Emsley and Bodenhausen 1992) and duration of 240.0 μs and 192.0 μs at 800 MHz, respectively. The adiabatic 180° Chirp broadband inversion pulse, denoted with striped half ellipsoid in both ^13^C channels, for inverting ^13^C^α^ and ^13^C′ magnetization in the middle of t_2_ period had duration of 500 μs at 800 MHz (Böhlen and Bodenhausen 1993). The Waltz-65 sequence (Zhou et al. 2007) with strength of 4.17 kHz was employed to decouple ^1^H spins. The GARP (Shaka et al. 1985, 1987) with field strength of 4.55 kHz was used to decouple ^13^C during acquisition. Delay durations: τ = 1/(4J_HC_) ~ 1.7 ms; τ_2_ = 3.4 ms (optimized for non-glycine residues) or 2.2–2.6 ms (for observing both glycine and non-glycine residues); ε = duration of G_H_ + field recovery ~ 1.2 ms; 2T_C_ = 1/(2J_CαC’_) ~ 9.5 ms; 2T_CAN_ ~ 28 ms. Maximum t_3_ is restrained t_3,max_ < 2.0*(T_CAN_–τ_2_). Frequency discrimination in ^13^C′ and ^15^N dimensions is obtained using the States-TPPI protocol (Marion et al. 1989) applied to ϕ_1_ and ϕ_2_, respectively, whereas the quadrature detection in ^13^C^α^ dimension is obtained using the sensitivity-enhanced gradient selection (Kay et al. 1992; Schleucher et al. 1994). The echo and antiecho signals in ^13^C^α^ dimension are collected separately by inverting the sign of the G_C_ gradient pulse together with the inversion of ψ, respectively. Phase cycling: ϕ_1_ = x, − x; ϕ_2_ = 2(x), 2(− x); ϕ_3_ = 4(y), 4(− y); ϕ_4_ = y; ψ = x; rec. = x, 2(− x), x, − x, 2(x), − x. Gradient strengths (% of max G/cm) and durations (ms): G_1_ = 17%, 0.234 ms; G_2_ = 40%, 1.0 ms; G_3_ = 60%, 1.0 ms; G_4_ = 25%, 1.0 ms; G_5_ = 35.7%, 0.234 ms; G_6_ = 7.1%, 0.234 ms; G_C_ = 80.0%, 1.0 ms; G_H_ = 20.1% 1.0 ms. The pulse sequences’ code and parameter file for Bruker Avance system are available from authors upon request

The magnetization transfer pathway of the new experiment we dubbed HACANCOi is shown in Fig. 1a. In brief, magnetization is first transferred from ^1^H^α^(*i*) to ^13^C^α^(*i*) and then simultaneously to ^13^C’(*i*) and ^15^N(*i*), as well as to ^15^ N(*i* + 1). The proposed 4D HACANCOi experiment is shown in Fig. 1b, whereas the inset b′ shows the alternative implementation for the 3D HA(CA)NCOi scheme. The coherence flows through the 4D HACANCOi, and 3D HA(CA)NCOi, experiment are as follows (Eqs. 1, 2):

1 $${}^{1}H^{\alpha } \left( i \right)\mathop{\longrightarrow}\limits^{{2\tau \left( {{}^{1}J_{H\alpha C\alpha } } \right)}}{}^{13}C^{\alpha } \left( i \right)\mathop{\longrightarrow}\limits^{{2T_{CAN} ,2T_{C} ,\tau_{2} \left( {{}^{1}J_{C\alpha N} ,{}^{2}J_{C\alpha N} ,{}^{1}J_{C\alpha C^{\prime}} ,{}^{1}J_{H\alpha C\alpha } } \right)}}{}^{13}C^{\prime}\left( i \right)\left[ {t_{1} } \right] \to^{15} N\left( {i,i + 1} \right)\left[ {t_{2} } \right] \to^{13}\!C^{\alpha } \left( i \right){{\left[ {2T_{CAN} - t_{3} ,\tau_{2} ;^{1} J_{C\alpha N} ,^{2} J_{C\alpha N} ,^{1} J_{{C\alpha C^{\prime}}} {^{1} J_{H\alpha C\alpha }} } \right]}}\mathop{\longrightarrow}\limits^{{4\tau \left( {^{1} J_{H\alpha C\alpha } } \right)}}{}^{1}H^{\alpha } \left( i \right)\left[ {t_{4} } \right],$$

2 $${}^{1}H^{\alpha } \left( i \right)\mathop{\longrightarrow}\limits^{{2\tau \left( {^{1} J_{H\alpha C\alpha } } \right)}}{}^{13}C^{\alpha } \left( i \right)\mathop{\longrightarrow}\limits^{{2T_{CAN} ,2T_{C} ,\tau_{2} \left( {^{1} J_{C\alpha N} ,^{2} J_{C\alpha N} ,^{1} J_{{C\alpha C^{\prime}}} ,^{1} J_{H\alpha C\alpha } } \right)}}{}^{13}C^{\prime}\left( i \right)\left[ {t_{1} } \right] \to^{15} N\left( {i,i + 1} \right)\left[ {t_{2} } \right] \to^{13}\!C^{\alpha } \left( i \right)\mathop{\longrightarrow}\limits^{{2T_{CAN} ,2T_{C} ,\tau_{2} \left( {^{1} J_{C\alpha N} ,^{2} J_{C\alpha N} ,^{1} J_{{C\alpha C^{\prime}}} ,^{1} J_{H\alpha C\alpha } } \right)}}\mathop{\longrightarrow}\limits^{{4\tau \left( {^{1} J_{H\alpha C\alpha } } \right)}}{}^{1}H^{\alpha } \left( i \right)\left[ {t_{3} } \right]$$

The experiments start with the ^1^H^α^(*i*) → ^13^C^α^(*i*) transfer, and the density operator immediately after the ϕ_4_ pulse is described as (time point *a*)

3 $$\sigma \left( a \right) = H_{z}^{\alpha } \left( i \right)C_{z}^{\alpha } \left( i \right)$$

Next, the magnetization is simultaneously transferred to both ^15^N(*i*) and ^15^N(*i* + 1) spins as well as to the ^13^C′(*i*) spin using ^13^C^α^–^15^N and ^13^C^α^–^13^C′ INEPT schemes, while refocusing the antiphase coherence with respect to the ^1^H^α^ spin. The composite pulse decoupling is then applied to remove the scalar interaction between ^1^H^α^ and ^13^C^α^ spins during the long transfer from the ^13^C^α^ to ^15^N spin. The density operator at time point *b* is

4 $$\sigma \left( b \right) = \left[ {C_{z}^{\alpha } \left( i \right)C^{\prime}_{z} \left( i \right)N_{z} \left( i \right)\Gamma_{2} + C_{z}^{\alpha } \left( i \right)C_{z}^{\prime } \left( i \right)N_{z} (i + 1)\Gamma_{3} } \right]\Gamma_{1} ,$$

where ${\Gamma }_{1}$ = sin(2π^1^*J*_CαC’_T_C_)cos^m^(2π^1^*J*_CαCβ_T_CAN_)sin(2π^1^*J*_CαHα_τ_2_)cos^n−1^(2π^1^*J*_CαHα_τ_2_), ${\Gamma }_{2}$ = sin(2π^1^*J*_NCα_T_CAN_)cos(2π^2^*J*_NCα_T_CAN_) and ${\Gamma }_{3}$ = cos(2π^1^*J*_NCα_T_CAN_)sin(2π^2^*J*_NCα_T_CAN_) (m equals 0 for glycines and 1 for other residues, n is the number of protons attached to the α-carbon i.e. 1 for non-glycine residues and 2 for glycines). This is followed by labeling of the ^13^C’ chemical shift in t_1_ and ^15^ N chemical shift in t_2_. Thus, the relevant density operator after the ϕ_3_ at the time point *c* is

5 $$\sigma \left( c \right) = \left[ {\begin{array}{*{20}c} {C_{x}^{\alpha } \left( i \right)C^{\prime}_{z} \left( i \right)N_{z} \left( i \right)\cos \left( {\omega_{N\left( i \right)} t_{2} } \right)\Gamma_{2} + } \\ {C_{x}^{\alpha } \left( i \right)C^{\prime}_{z} \left( i \right)N_{z} \left( {i + 1} \right)\cos \left( {\omega_{{N\left( {i + 1} \right)}} t_{2} } \right)\Gamma_{3} } \\ \end{array} } \right]\Gamma_{1} \cos \left( {\omega_{C^{\prime}\left( i \right)} t_{1} } \right)$$

During the ensuing delay 2*T_CAN_, the desired coherence is refocused with respect to ^15^N and ^13^C′ spins and antiphase coherence with respect to the ^1^H^α^ is generated during τ_2_. In the 4D HACANCOi experiment, this period is used for labeling of the chemical shift of ^13^C^α^ in a constant-time manner. Therefore, the relevant density operator prior to the coherence order selective coherence transfer (COS-CT) step is (time point *d*)

6 $$\sigma \left( d \right) = \left[ {\begin{array}{*{20}c} {H_{z}^{\alpha } \left( i \right)C^{\alpha - } \left( i \right)\cos \left( {\omega_{N\left( i \right)} t_{2} } \right)\Gamma_{2}^{2} + } \\ {H_{z}^{\alpha } \left( i \right)C^{\alpha - } \left( i \right)\cos \left( {\omega_{{N\left( {i + 1} \right)}} t_{2} } \right)\Gamma_{3}^{2} } \\ \end{array} } \right]\Gamma_{1}^{2} \cos \left( {\omega_{{C^{\prime}\left( i \right)}} t_{1} } \right)\exp \left( { \pm \omega_{C\alpha \left( i \right)} t_{3} } \right)$$

For the 3D HA(CA)NCO the back-transfer step (inset b′ to Fig. 1) deviates slightly as no chemical shift of ^13^C^α^ is labeled during t_3_. Finally, COS-CT with gradient echo (Kay et al. 1992; Schleucher et al. 1994) is used to transfer the magnetization back to ^1^H^α^ and the signal of interest corresponds to the density operator at the time point *e*

7 $$\sigma \left( e \right) = \left[ {\begin{array}{*{20}c} {H^{\alpha - } \left( i \right)\cos \left( {\omega_{N\left( i \right)} t_{2} } \right)\Gamma_{2}^{2} + } \\ {H^{\alpha - } \left( i \right)\cos \left( {\omega_{{N\left( {i + 1} \right)}} t_{2} } \right)\Gamma_{3}^{2} } \\ \end{array} } \right]\Gamma_{1}^{2} \cos \left( {\omega_{{C^{\prime}\left( i \right)}} t_{1} } \right)\exp \left( { \pm i\omega_{C\alpha \left( i \right)} t_{3} } \right)\exp \left( {i\omega_{H\alpha \left( i \right)} t_{4} } \right)$$

Hence, the 4D HACANCOi experiment shows *intraresidual* cross peaks at ω_Hα(i)_, ω_Cα(i)_, ω_N(i),_ ω_C’(i)_, and *sequential* correlations at ω_Hα(i)_, ω_Cα(i)_, ω_N(i+1),_ ω_C’(i)_, frequencies, respectively. Analogously the 3D HA(CA)NCOi experiment exhibits *intraresidual* correlations at ω_Hα(i)_, ω_N(i),_ ω_C’(i)_, and *sequential* correlations at ω_Hα(i)_, ω_N(i+1),_ ω_C’(i)_ coordinates. Of note, given the frequency labeling of the ^13^C^α^ chemical shift during t_3_ takes place in a constant-time manner and employs sensitivity enhanced gradient echo during t_3_, there is no dimensionality-associated sensitivity loss between the 3D and 4D versions of the experiment.

To minimize adjustments of sample conditions and maximize availability of structural restraints from one sample, that is, H^N^-based distance restraints and ^15^N spin relaxation rates for instance, we prefer measuring the ^1^H^α^-detected experiments in 95%/5% H_2_O/D_2_O. This demands a relatively robust water suppression as H^α^ spins often resonate very close to the residual water signal. To meet these requirements, the water flip-back approach together with the sensitivity-enhanced heteronuclear gradient-echo implementation is employed. Therefore, the bulk water magnetization is spin-locked throughout most of the experiment and residual transversal water magnetization components are dephased by the pulsed field gradients. In our hands this approach works sufficiently as shown for the first increment of the HACANCOi experiment from ^15^N,^13^C EspF: unlabeled SNX9-SH3 complex (Supplementary Fig. 1). Water suppression can be further optimized by adjusting the length of the gradient pulse G_3_ (1–4 ms) between the t_1_ and t_2_ evolution periods. Nevertheless, if the protein concentration is low, < 0.3 mM, use of pure D_2_O as a solvent is worth considering.

### Coherence transfer efficiency of HACANCOi

The coherence transfer efficiency for the intraresidual and interresidual correlations in the 4D HACANCOi scheme can be calculated according to Eqs. 8 and 9:

8 $$I_{{HACANCOi\left( {{\text{intra}}} \right)}} = \Gamma_{1}^{2} \Gamma_{2}^{2} \exp \left( { - 2T_{CAN} /T_{2,C\alpha } } \right),$$

9 $$I_{{HACANCOi\left( {sequential} \right)}} = \Gamma_{1}^{2} \Gamma_{3}^{2} \exp \left( { - 2T_{CAN} /T_{2,C\alpha } } \right).$$

**Fig. 2:**
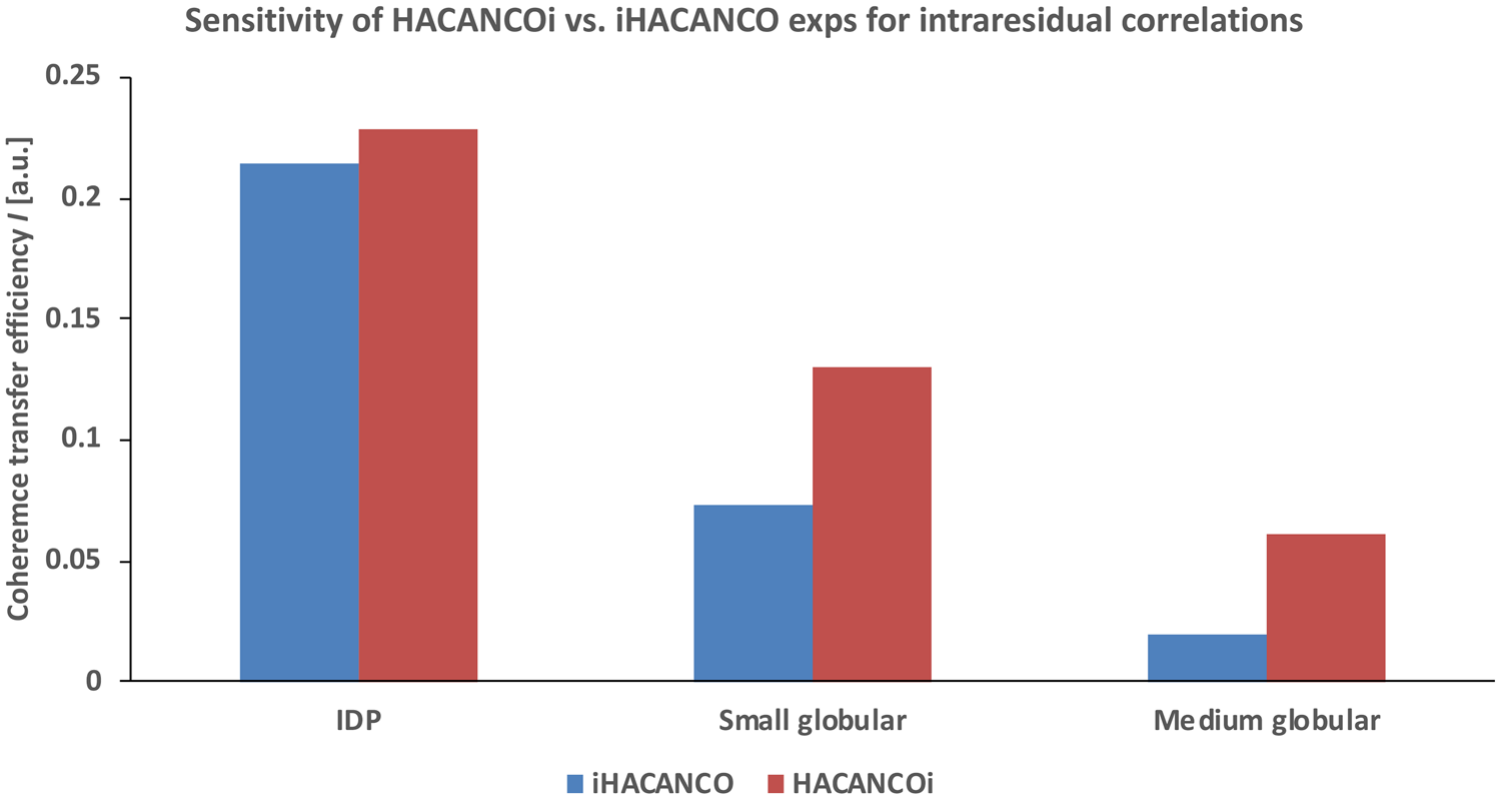
Comparison of theoretical coherence transfer efficiencies for the HACANCOi and the intraresidual iHACANCO experiments. Calculations were carried out for three proteins having diverging T_2_ relaxation properties. The bars on the left hand side represent the coherence transfer efficiency for a typical IDP, having T_2_ relaxation times of 100, 200 and 200 ms for ^13^C^α^, ^13^C′ and ^15^N, respectively. These values are significantly shorter for small and (medium sized) globular protein, i.e. 50 (30), 100 (60) and 100 (60) ms. For calculations, we assumed ^1^J_CαC′_ = 53 Hz, ^1^J_C′N_ = 15 Hz and ^1^J_CαCβ_ = 35 Hz. For an IDP, random coil conformation with ^1^J_NCα_ = 10.6 Hz and ^2^J_NCα_ = 7.5 Hz values is presumed. For small and medium sized structural proteins, α-helical conformation is assumed with 9.6 Hz and 6.4 Hz scalar interactions for one- and two-bond couplings between ^13^C^α^ and ^15^N, respectively (Delaglio et al. 1991)

Assuming a transverse relaxation time of 100 ms for the ^13^C^α^ spin in an IDP, the coherence transfer efficiency *I* for the first increment is 0.23 (0.08) for the intraresidual (sequential) pathway. For small (*T*_*2,C*α_ = 50 ms) and medium (*T*_*2,C*α_ = 30 ms) sized proteins, having residues in an α-helical conformation, the transfer efficiency becomes 0.13 (0.04) and 0.06 (0.02), respectively. In these calculations, we set ^1^*J*_CαC’_ = 53 Hz, ^1^*J*_C’N_ = 15 Hz and ^1^*J*_CαCβ_ = 35 Hz. In addition, ^1^*J*_NCα_ and ^2^*J*_NCα_ values in random coil (α-helical) conformation are assumed to be 10.6 (9.6) Hz and 7.5 (6.4) Hz, respectively (Delaglio et al. 1991). We have neglected one-bond ^1^*J*_CαHα_ coupling and ^1^H^α^ transverse relaxation times in these calculations since the pulse sequences compared here and those previously shown, have a similar implementation for ^1^H–^13^C transfer. In order to maximize the coherence transfer efficiency for the intraresidual pathway, the delay 2T_CAN_ should be set to 28 ms. If we calculate the theoretical coherence transfer efficiencies and compare the performance of the proposed 4D HACANCOi experiment with the 4D iHACANCO experiment (Tossavainen et al. 2020), we observe that for the assignment of IDPs, the two experiments offer similar sensitivity, *I*_HACANCOi_ = 0.228 and *I*_iHACANCO_ = 0.214, as can be seen in Fig. 2. However, the intraresidual 4D iHACANCO scheme lifts the data redundancy problem by displaying solely the intraresidual cross peaks (Mäntylahti et al. 2010; Tossavainen et al. 2020). Remarkably, for the assignment of small and medium sized globular proteins, in which the mobility of the polypeptide chain, and hence the transverse relaxation time, is dominated by the overall rotational tumbling, the proposed 4D HACANCOi experiment clearly outperforms the 4D iHACANCO. This is exemplified in Fig. 2, which shows that the transfer efficiency of the new 4D HACANCOi experiment is almost two-fold higher than that of the 4D iHACANCO experiment in small globular proteins. The difference is even more pronounced in the case of medium sized globular proteins, approaching a three times higher throughput for the new experiment.

### Comparison of attainable sensitivities between the HACANCOi and iHACANCO experiments

**Fig. 3:**
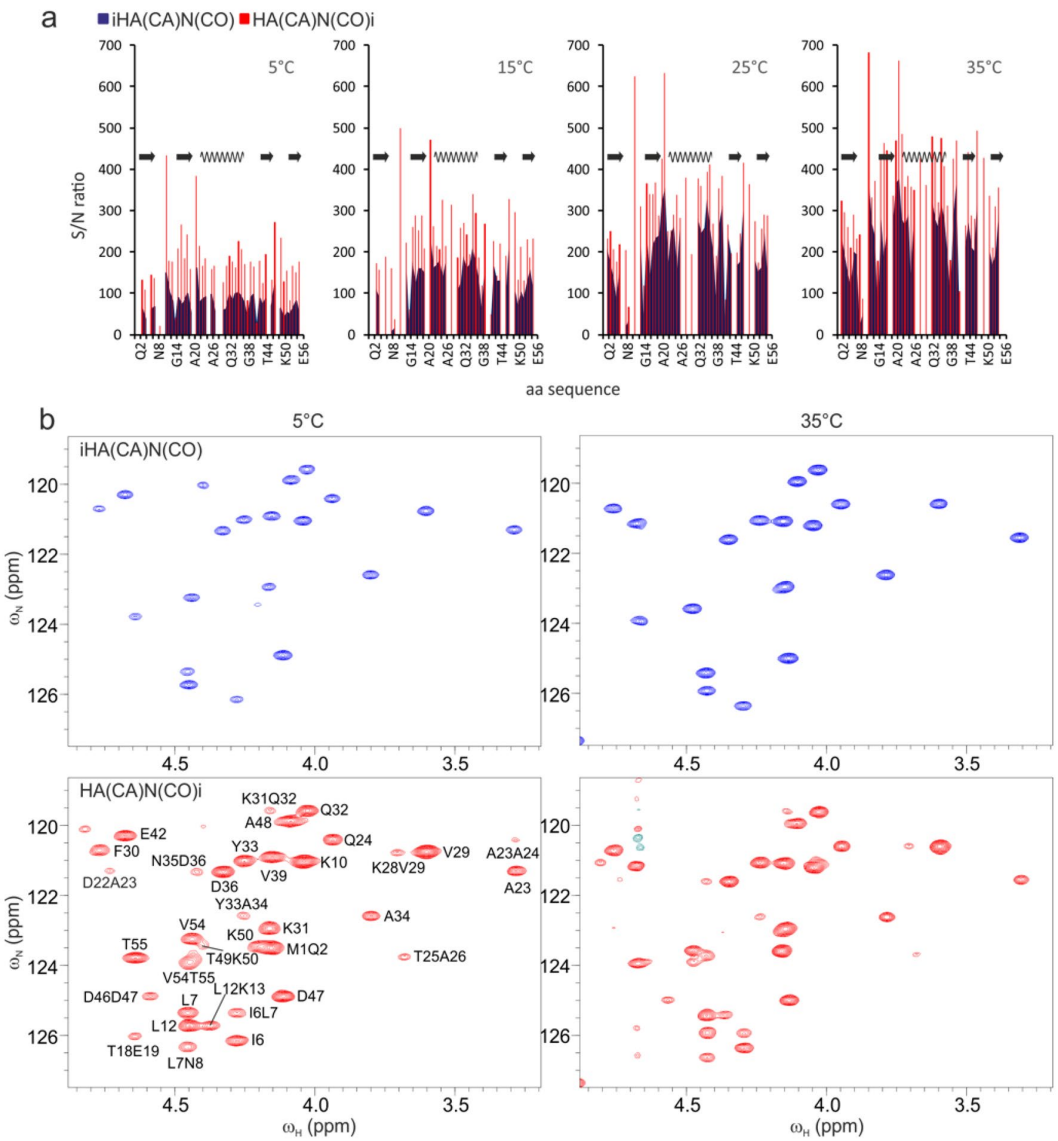
Comparison of sensitivities between the proposed HACANCOi and the intraresidual iHACANCO experiments. **a** Bar graphs of per residue signal to noise ratios in the two experiments at four temperatures. The horizontal axis shows the amino acid sequence. On top of the panels is shown the secondary structure as present in the 25 °C NMR solution structure of GB1 (PDB ID 2GB1, Gronenborn et al. 1991), arrows representing β-strands and the wave an α-helix. Measurements for the S/N ratios were carried out at four different temperatures, 5, 15, 25, and 35 °C. The ratios were measured from two-dimensional ^1^H^α^, ^15^N correlation spectra of iHA(CA)N(CO) and HA(CA)N(CO)i experiments. For glycine two ^1^H^α^s, if not degenerate, the average of the ratios is shown. Due to overlap with the residual water signal or with an interresidual peak some of the bars are missing from the graph. The bar of E56 is not shown due to the 90º phase difference in the signal of the C-terminal residue, and thereby meaningless comparison (Mäntylahti et al. 2010). **b** Expansions of GB1 iHA(CA)N(CO) and HA(CA)N(CO)i spectra at 5 and 35 °C. Peak assignment is shown in the 5 °C HA(CA)N(CO)i spectrum for both the i and i + 1 peaks. The base level of iHA(CA)N(CO) and HA(CA)N(CO)i experiments at each temperature is identical i.e. the peak intensities are directly comparable between the two experiments

For experimental verification of the coherence transfer efficiency in the new 4D HACANCOi experiment, we next compared the attainable intensities for the intraresidual cross peaks between the two two-dimensional experiments, i.e. the 2D implementation of the proposed HA(CA)N(COi) and the 2D iHA(CA)N(CO) pulse schemes (Mäntylahti et al. 2010). As can be recognized from Fig. 3, showing the per residue signal to noise ratio for the small B1 domain of protein G (GB1, molecular weight 6.6 kDa) between the two experiments, the sensitivity of the HACANCOi experiment is superior with respect to iHACANCO in all measured temperatures. On average, the largest gain in sensitivity is observed for the largest molecule, simulated with lowering the GB1 sample temperature to 5 ºC. The correlation times of GB1 at 5, 15, 25, and 35 °C are 7.5, 5.4, 4.1 and 3.2 ns, which further correspond to molecular weights of 13, 9, 7 and 5 kDa, respectively. The sensitivity gain in loop regions is somewhat more pronounced.

The sensitivity enhancement scheme with the gradient echo warrants high quality water suppression, but is likely to become inefficient for high molecular weight complexes beyond 20 kDa. To this end, the conventional back-INEPT transfer from ^13^C^α^ to ^1^H^α^ might be an advantageous implementation (See Supplementary Material, Fig. S2).

### Application of 3D HA(CA)NCOi and 4D HACANCOi pulse schemes for the assignment of EPEC EspF–SNX9 SH3 complex

**Fig. 4:**
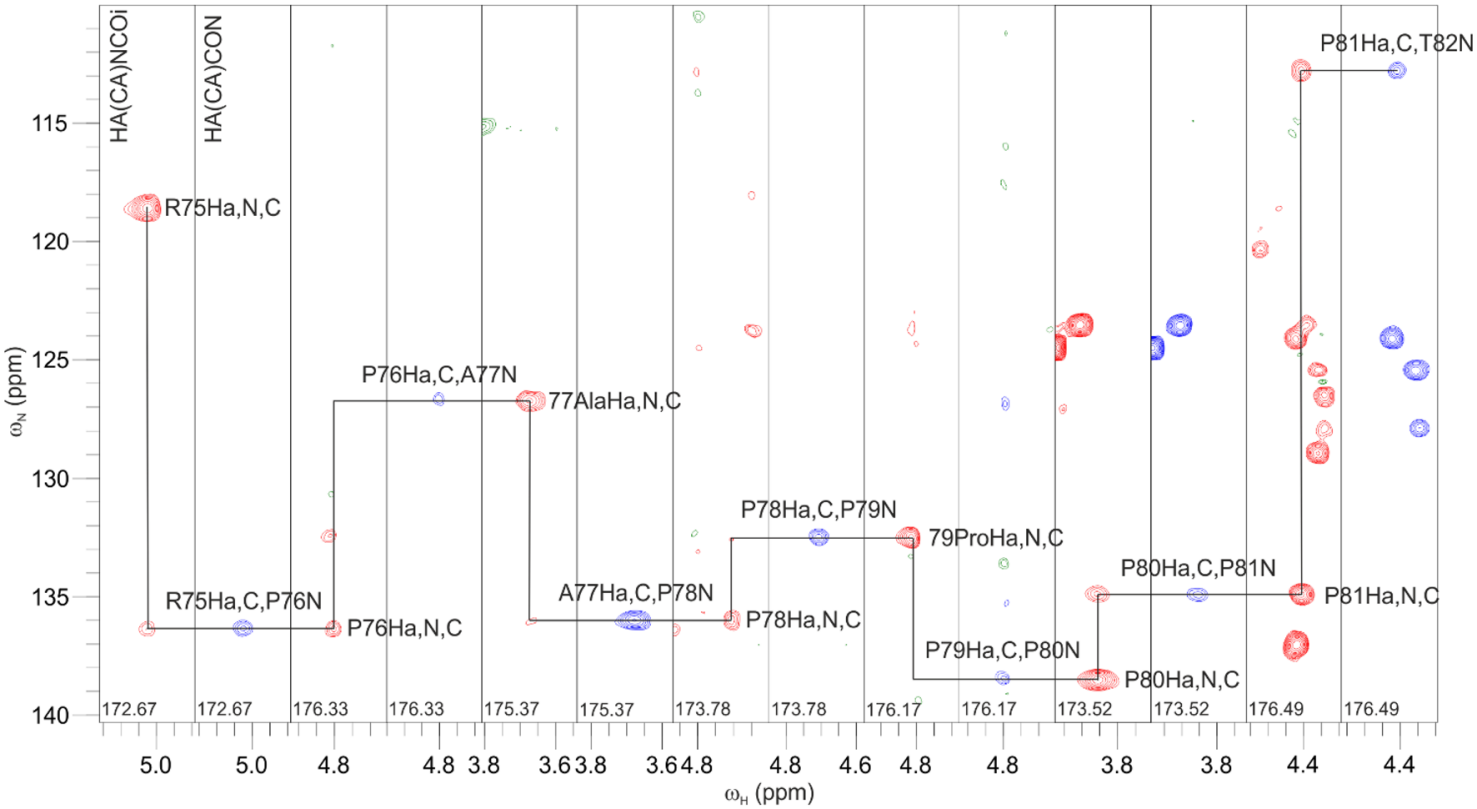
Representative strip plots from the proposed 3D HA(CA)NCOi (left panel, red contours) and 3D HA(CA)CON (right panel, blue contours) experiments from the proline-rich region ^75^RPAPPPP^81^ of ^15^N, ^13^C labeled EspF in complex with unlabeled SNX9 SH3. In the HA(CA)NCOi experiment strong intraresidual ω_Hα(i)_–ω_C′(i)_–ω_N(i)_ correlations are observed together with weaker (or absent) sequential ω_Hα(i)_–ω_C′(i)_–ω_N(i+1)_ correlations

To test the applicability of the novel 3D HA(CA)NCOi and 4D HACANCOi experiments, we employed them for the sequential assignment of the enteropathogenic *E.* *coli* (EPEC) EspF–SNX9 SH3 complex. SH3 domains are ubiquitous structural modules that typically recognize proline-rich linear motifs, harboured in IDPs/IDRs (Saksela and Permi 2012). This motif in our target, the second 47-residue repeat of EspF, contains five prolines in a stretch of seven residues. Figure 4 displays strip plots of 3D HA(CA)NCOi and 3D HA(CA)CON (Mäntylahti et al. 2010) spectra of this stretch collected from a sample composed of ^15^N, ^13^C labeled EspF bound to unlabeled SNX9 SH3. It exemplifies the robustness of the H^α^-detection strategy for establishing sequential connectivities through proline-rich regions Indeed, the “sequential walk” through multiple consecutive proline residues, located at the EspF–SH3 binding interface, was successful with spectra measured from a sample dissolved in 95%/5% H_2_O/D_2_O. This is remarkable considering that three of the prolines’ H^α^ shifts nearly coincide with that of the residual water signal. Use of ^1^H^α^, ^13^C’ and ^15^N chemical shifts warranted good dispersion of signals and full backbone resonance assignment of bound EPEC EspF was obtained. All expected *intra-* and *interresidual* peaks were present in the HA(CA)NCOi and HA(CA)CON spectra, respectively.

**Fig. 5:**
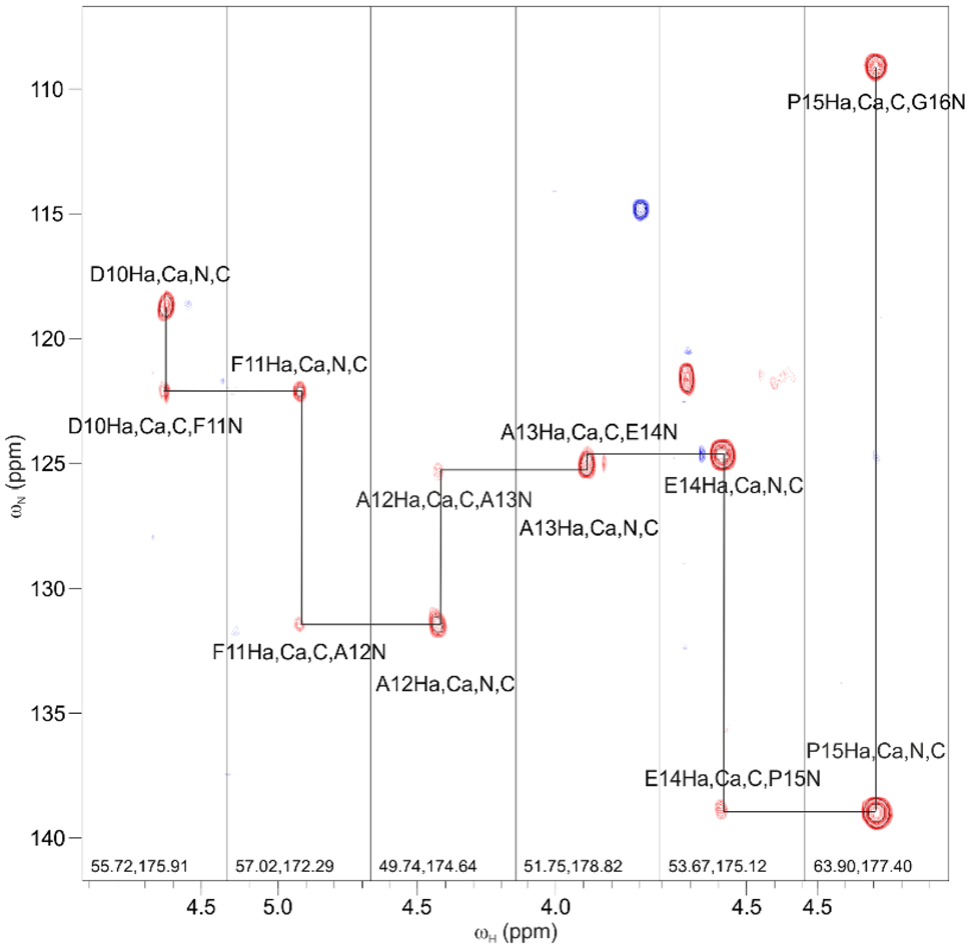
Strip plots of the proposed 4D HACANCOi experiment. Spectra display the “sequential walk” through amino acid residue stretch ^10^DFAAEP^15^ in ^15^N, ^13^C labeled SNX9 SH3 in complex with unlabeled EPEC EspF. This stretch forms a part of the EPEC EspF binding site on SNX9 SH3. Both intraresidual ^1^H^α^(i), ^13^C^α^(i), ^13^C′(i), ^15^N(i) and sequential ^1^H^α^(i), ^13^C^α^(i), ^13^C’(i), ^15^N(i + 1) correlations are observed, allowing sequential assignment of ^1^H^α^, ^13^C^α^, ^13^C′ and ^15^N backbone resonances. Although in the example given here, 4D HACANCOi displayed both intra- and interresidual correlations, usually a complementary 4D HACACON experiment (Tossavainen et al. 2020) is needed to confirm interresidual connectivities

For the assignment of ^1^H, ^13^C, ^15^N backbone resonances of SNX9 SH3, we prepared a reversibly labeled complex, that is ^15^N, ^13^C labeled SNX9 SH3 was bound to unlabeled EPEC EspF. In order to link the ^13^C^α^ chemical shifts directly to ^1^H^α^ resonances and to obtain additional signal dispersion without compromising the overall sensitivity, we employed the 4D HACANCOi experiment. Figure 5 shows an excerpt of 4D HACANCOi strip plots in which the intraresidual ^15^ N(*i*) and interresidual ^15^ N(*i* + 1) chemical shifts are correlated with their corresponding ^1^H^α^(*i*), ^13^C′(*i*) and ^13^C^α^(*i*) chemical shifts. This provides highly dispersed resonance correlation maps, but also readily offers ^1^H^α^, ^13^C^α^, and ^13^C’ chemical shift data, which are the most useful spins for calculating transiently populated secondary structures in IDPs as they are particularly sensitive to the ϕ/ψ angles of the protein backbone (Borcherds and Daughdrill 2018). Except for two residues whose H^α^ resonances are very close to that of residual water, all expected *intra* peaks were present in the 4D spectrum.

Assignment of disordered proteins confronts the well-established H^N^-detected triple-resonance approaches due to clustering of ^13^C^α^/^13^C^β^ chemical shifts by residue type, susceptibility of H^N^ resonances to chemical exchange broadening at alkali or neutral pH, and also high abundance of prolines in IDPs (Mäntylahti et al. 2010). To circumvent problems associated with the H^N^-detection on IDPs, the ^13^C-detection strategy has been employed successfully. It does not suffer from the exchange broadening of signals and yet allows assignment of prolines. Like H^N^-detected experiments they often apply the ‘CON’ strategy for connecting neighboring residues with highly degenerate chemical shifts (Pantoja-Uceda and Santoro 2014; Sahu et al. 2014; Brutscher et al. 2015; Chaves-Arquero et al. 2018). Another salient feature of the ^13^C-detected strategy is the absence of the disturbing residual water signal, i.e. no special care needs to be undertaken for water handling in these experiments. Further improvements in resolution and sensitivity stem from the fact that two- and three-bond ^13^C–^13^C couplings are much smaller in size than the corresponding ^1^H–^1^H couplings. The caveat of this strategy is the inherently lower sensitivity of ^13^C-detection, by a factor of 8, with respect to ^1^H-detection. This can be partially compensated (by a factor of ca. 2) by using a triple-resonance probehead with ^13^C as the inner coil. We have proposed using ^1^H^α^-detection as an alternative to the N^H^- or ^13^C-detection strategies (Mäntylahti et al. 2010, 2011). Indeed, ^1^H^α^-detection offers the best of both worlds, higher sensitivity of ^1^H-detection in general and the possibility to work on IDPs under alkali conditions and/or abundant proline content. Thus far our assignment strategy has followed solely unidirectional coherence transfer routes. That is, active purging of redundant information between spectra using so-called *intraresidual* (Brutscher 2002; Permi 2002) or *sequential* coherence transfer was employed in the 3D and 4D iHACANCO, HACACON and (HACA)CONCAHA experiments (Mäntylahti et al. 2010, 2011; Tossavainen et al. 2020). In this paper, we have introduced the novel HACANCOi experiment that offers superior sensitivity for the assignment of IDPs which establish a slowly dissociating complex with their binding partner thus rendering their relaxation properties less favorable towards more selective pulse sequences. The new experiment utilizes a superior ^15^N–^13^C′ correlation map to maximize chemical shift dispersion in challenging IDP/IDRs (Yao et al. 1997; Mäntylahti et al. 2009; Bermel et al. 2012), although both *i* and *i* + 1 correlations are visible in the ^15^N dimension. The coherence transfer efficiency is clearly superior to the corresponding intraresidual only iHACANCO experiment (Mäntylahti et al. 2010; Tossavainen et al. 2020) even for the smallest globular protein or complex. Recently, Wong et al. proposed a suite of H^α−^ detected experiments, including e.g., haCONHA and haNCOHA that yield H^α^(*i*), C′(*i*), N(*i* + 1), and H^α^(*i* + 1), C′(*i*), N(*i* + 1) (as well as H^α^(i), C′(*i*), N(*i* + 1)) correlations, respectively (Wong et al. 2020). They found haNCONHA to be on average 3.5 times more sensitive than the ^13^C-detected counterpart, haCON. Our proposed HACANCOi experiment, based on the calculation shown above, has 1.9 and 2.8 times higher theoretical S/N for small and medium sized proteins/complexes, respectively, compared to that of haNCOHA. This stems from the fact that in HACANCOi, magnetization is transferred simultaneously from the ^13^C^α^(*i*) spin to ^13^C′(*i*) and to ^15^N(*i*)/^15^N(*i* + 1) spins during the delay 2T_CAN_. The experiment is then conceptually similar to the H^N^-detected HNCO,CA TROSY experiment proposed by Konrat et al. (1999) for the assignment of high-molecular weight proteins.

In case of high molecular weight complexes beyond 20 kDa, the sensitivity-enhanced Rance-Kay scheme may become inefficient in comparison to the conventional back-INEPT transfer. To this end, the version of the pulse sequence without the sensitivity-enhanced gradient selection shown in the Supplementary material should be employed (Fig. S2). In such a case, it may be necessary to dissolve the sample in pure D_2_O instead of H_2_O/D_2_O for sufficient water suppression. Distinguishing glycine residues from other residues is not as straightforward as in the iHACANCO experiment since ${\Gamma }_{1}$ contains a cos^m^(2π^2^*J*_CαCβ_T_CAN_) term, where 2T_CAN_ is set to 28 ms. Therefore, separation of glycines based on their 180° difference in sign is not possible with the proposed experiment due to ${\Gamma }_{1}^{2}$ dependence. However, extending the first τ_2_ delay to 4.4 ms yields a 180° phase inversion for glycines, if deemed necessary (Mäntylahti et al. 2009).

It is noteworthy that the proposed HACANCOi experiment as well as the iHACANCO and HACACON (Mäntylahti et al. 2010; Tossavainen et al. 2020) schemes are optimal for proline assignment from the coherence transfer efficiency point of view. The magnetization is transferred direcly from the ^13^C^α^(*i*) spin to the ^15^N(*i*) spin of proline. Indeed, being an N-substituted residue, the ^15^N spin of proline exhibits ^1^*J*_NCα_ and ^1^*J*_NCδ_ couplings of similar size and hence the *straight-through* ^1^H → ^13^C → ^15^ N → ^13^C → ^1^H type of experiments, such as (HACA)CONCAH (Mäntylahti et al. 2011; Yao et al. 2014; Tossavainen et al. 2020) are less sensitive than the *out-and-back* (^1^H ←  → ^13^C ←  → ^15^N) type experiments shown here.

## Conclusions

We have introduced a new NMR experiment, HACANCOi, using ^1^H^α^-detection for the assignment of backbone ^1^H^α^_i_, ^13^C^α^_i_, ^15^N_i_ and ^13^C′_i_ resonances in ^15^N, ^13^C labeled proteins. In comparison to the established intraresidual iHACANCO experiment (Mäntylahti et al. 2010; Tossavainen et al. 2020), the novel experiment yields two cross peaks per residue and hence suboptimal resolution, but offers superior sensitivity which may become a limiting factor in the assignment of IDPs forming a complex with a more slowly tumbling structural protein. As we have demonstrated here, the new experiment thrives in molecular systems established by a disordered region bound to a globular domain in which the attainable transverse relaxation rates resemble those of structural proteins rather than IDPs with highly elevated dynamics. The new experiment further extends the possibilities of employing the ^1^H^α^-detection approach to systems enriched with proline residues and/or studied under alkali conditions where detection of amide protons becomes difficult.

## Electronic supplementary material

Below is the link to the electronic supplementary material.

Supplementary file1 (PDF 224 kb)
