# Supplementary material for: HACANCOi: a new Hα-detected experiment for backbone resonance assignment of intrinsically disordered proteins
Source: J Biomol NMR. 2020 Oct 28;74(12):741–52. doi: 10.1007/s10858-020-00347-5 (PMC7701164; doi:10.1007/s10858-020-00347-5)
Supplement: Supplementary file 1 — Supplementary file1 (PDF 224 kb) [file 10858_2020_347_MOESM1_ESM.pdf]

Supplementary material

HACANCOi: a new H^{α}-detected experiment for backbone resonance assignment of intrinsically disordered proteins

Mikael Karjalainen[1], Helena Tossavainen[2], Maarit Hellman[2], Perttu Permi[1,2*]

[1]Department of Chemistry, Nanoscience Center, University of Jyvaskyla, Jyvaskyla, Finland.
[2]Department of Biological and Environmental Science, University of Jyvaskyla, Jyvaskyla, Finland

*Corresponding author:
Perttu Permi, perttu.permi@jyu.fi

ORCID of the authors:
Mikael Karjalainen, 0000-0003-3154-7570
Helena Tossavainen, 0000-0002-1609-1651
Perttu Permi, 0000-0002-6281-1138

Fig. S1.

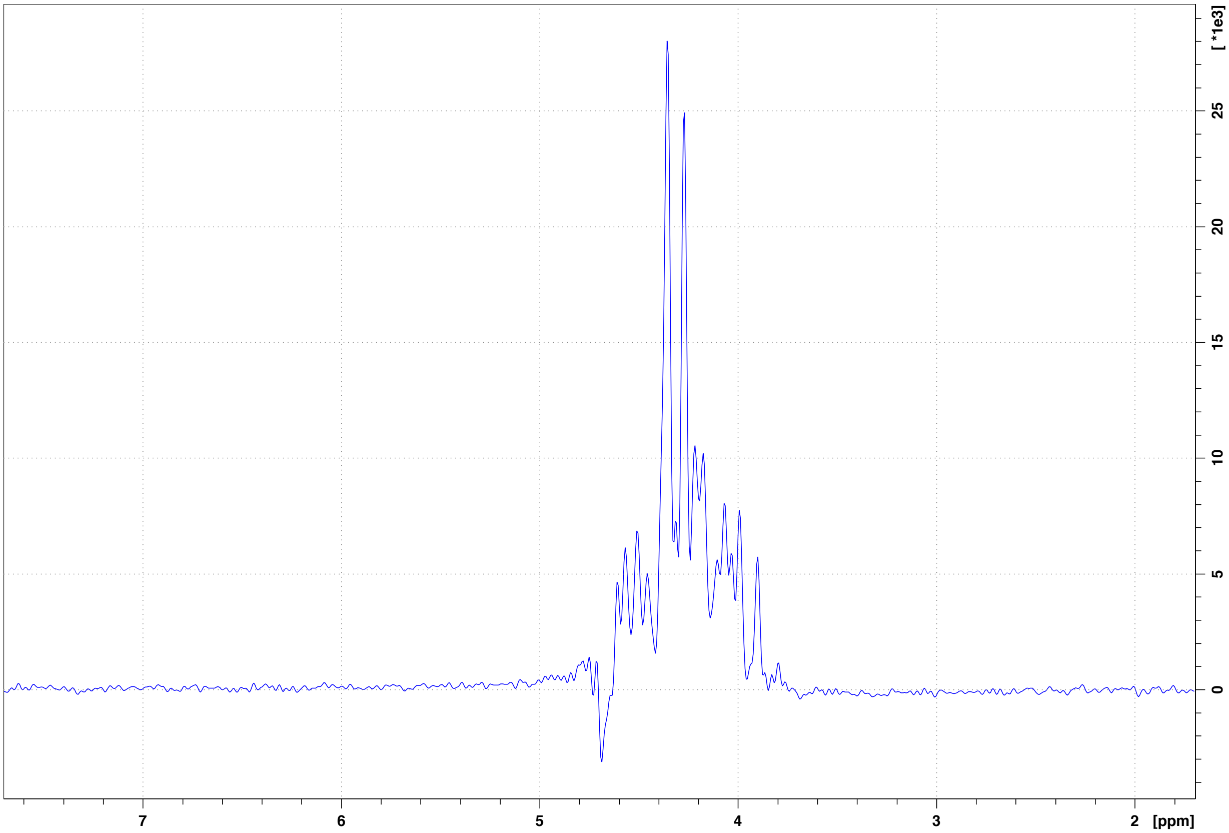

Fig. S1. The first increment of HA(CA)NCOi on ^{15}N,^{13}C labeled EspF in complex with unlabeled SNX9-SH3.

The sample was 1.5 mM ^{15}N,^{13}C labeled EspF: 2.5 mM unlabeled SNX9-SH3 complex in 95/5% H_2O/D_2O. The experiment was executed with 16 scans per FID and recycle delay of 0.85 seconds. No post-acquisition solvent suppression was employed prior to Fourier transform.

Fig. S2.

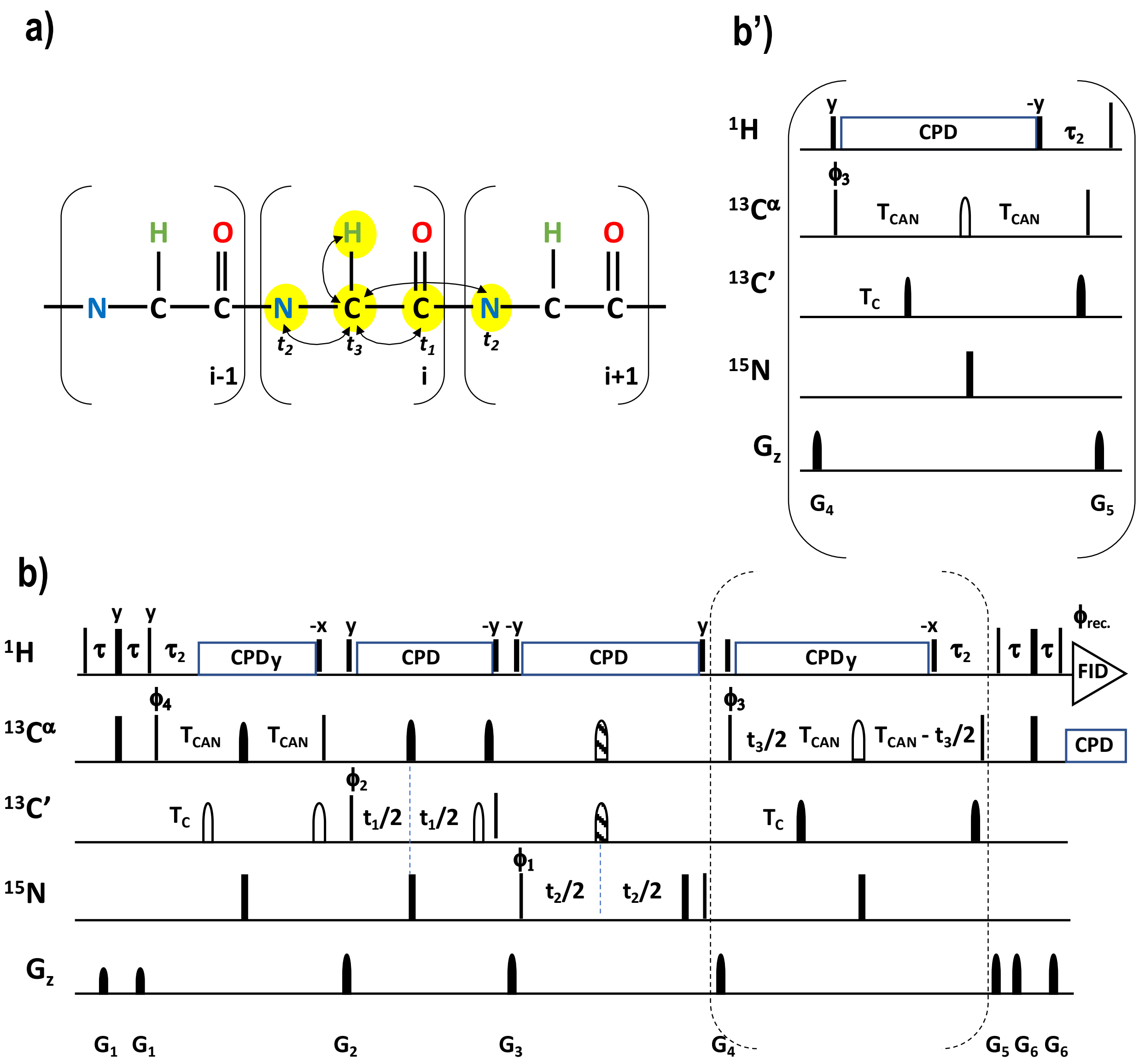

Fig. S2. Description of the HACANCOi experiment without the Rance-Kay sensitivity enhancement.

a) Schematic presentation of magnetization transfer pathway during the 4D HACANCOi experiment. Black arrows indicate the so-called out-and-back transfer pathway from $^{1}H^{\alpha}(i)$ to $^{13}C^{\alpha}(i)$ and further to $^{13}C'(i)$ and $^{15}N(i)/^{15}N(i+1)$. **b)** 4D HACANCOi experiment to correlate $^{1}H^{\alpha}(i)$, $^{13}C^{\alpha}(i)$, $^{13}C'(i)$ and $^{15}N(i)/^{15}N(i+1)$ chemical shifts. Inset **b')** 3D HA(CA)NCOi experiment to correlate $^{1}H^{a}(i)$, $^{13}C'(i)$ and $^{15}N(i)/^{15}N(i+1)$ chemical shifts. Narrow and wide filled bars on ^{1}H and ^{15}N channels correspond to rectangular 90° and 180° pulses, respectively, applied with phase x unless otherwise stated. All ^{13}C pulses are band-selective shaped pulses, denoted by

filled narrow bars (90°) and filled and unfilled half ellipsoids (180°). Pulses denoted with unfilled bars are applied on-resonance. The ^{1}H, ^{15}N, $^{13}C'$, and $^{13}C^{\alpha}$ carrier positions are 4.7 (water), 121 (center of ^{15}N spectral region), 174 ppm (center of $^{13}C'$ spectral region), and 54 ppm (center of $^{13}C^{\alpha}$ spectral region). The ^{13}C carrier is initially set to the middle of $^{13}C'$ region (174 ppm), and shifted to $^{13}C^{\alpha}$ region (54 ppm) prior to 90° ^{15}N pulse ϕ_3. All band-selective 90° and 180° pulses for $^{13}C^{\alpha}$ (54 ppm) and $^{13}C'$ (174 ppm) have the shape of Q5 and Q3 (Emsley and Bodenhausen 1992) and duration of 240.0 ms and 192.0 ms at 800 MHz, respectively. The adiabatic 180° Chirp broadband inversion pulse, denoted with striped half ellipsoid in both ^{13}C channels, for inverting $^{13}C^{a}$ and $^{13}C'$ magnetization in the middle of t_2 period had duration of 500 ms at 800 MHz (Böhlen and Bodenhausen 1993). The Waltz-65 sequence (Zhou et al. 2007) with strength of 4.17 kHz was employed to decouple ^{1}H spins. The GARP (Shaka et al. 1985, 1987) with field strength of 4.55 kHz was used to decouple ^{13}C during acquisition. Delay durations: $\tau = 1/(4J_{HC}) \sim 1.7$ ms; $\tau_2 = 3.4$ ms (optimized for non-glycine residues) or 2.2 - 2.6 ms (for observing both glycine and non-glycine residues); $2T_C = 1/(2J_{C^{\alpha}C'}) \sim 9.5$ ms; $2T_{CAN} \sim$ 28 ms. Maximum t_3 is restrained $t_{3,max} < 2.0*(T_{CAN} - t_2)$. Frequency discrimination in $^{13}C'$, ^{15}N and $^{13}C^{\alpha}$ dimensions is obtained using the States-TPPI protocol (Marion et al. 1989) applied to ϕ_1, ϕ_2, and ϕ_3, respectively. Phase cycling: ϕ_1 = x, -x; ϕ_2 = 2(x), 2(-x); ϕ_3 = 4(y), 4(-y); ϕ_4 = y; rec. = x, 2(-x), x, -x, 2(x), -x. Gradient strengths (% of max G/cm) and durations (ms): G_1 = 17 %, 0.234 ms; G_2 = 40 %, 1.0 ms; G_3 = 60 %, 1.0 ms; G_4 = 25 %, 1.0 ms; G_5 = 80 %, 1.0 ms; G_6 = 35.7 %, 0.234 ms.

References

Böhlen J-M, Bodenhausen G (1993) Experimental aspects of chirp NMR spectroscopy. J Magn Reson Ser A 102:293–301. doi: 10.1006/jmra.1993.1107

Emsley L, Bodenhausen G (1992) Optimization of shaped selective pulses for NMR using a quaternion description of their overall propagators. J Magn Reson 97:135–148. doi: 10.1016/0022-2364(92)90242-Y

Marion D, Ikura M, Tschudin R, Bax A (1989) Rapid recording of 2D NMR-spectra without phase cycling – application to the study of hydrogen-exchange in proteins. J Magn Reson 85:393-399. doi: 10.1016/0022-2364(89)90152-2

Shaka AJ, Barker PB, Freeman R (1985) Computer-optimized decoupling scheme for wideband applications and low-level operation. J Magn Reson 64:547–552. doi: 10.1016/0022-2364(85)90122-2

Shaka AJ, Keeler J (1987) Broadband spin decoupling in isotropic liquids. Prog Nucl Magn Reson Spectrosc 19:47–129. doi: 10.1016/0079-6565(87)80008-0

Zhou Z, Kümmerle R, Qiu X, Redwine D, Cong R, Taha A, Baugh D, Winniford B (2007) A new decoupling method for accurate quantification of polyethylene copolymer composition and triad sequence distribution with 13C NMR. J Magn Reson 187:225–233. doi: 10.1016/j.jmr.2007.05.005
